# Precision dentistry in preclinical education: analytic rubrics for enhanced objectivity in metal-ceramic crown preparations

**DOI:** 10.3389/fdmed.2025.1728355

**Published:** 2026-01-07

**Authors:** Saeed M. Alqahtani

**Affiliations:** Department of Prosthetic Dentistry, College of Dentistry, King Khalid University, Abha, Saudi Arabia

**Keywords:** crowns, precision dentistry, dental education, FPD, analytic rubric, preclinical assessment

## Abstract

**Introduction:**

Preclinical dental training requires students to master precise tooth preparation skills that directly influence clinical success. Traditional grading methods often lack objectivity and consistency. Analytic rubrics, by deconstructing complex procedures into measurable components, may improve reliability and feedback in preclinical assessment. This study evaluated the application of an analytic rubric system for assessing anterior metal-ceramic crown preparations performed by undergraduate dental students.

**Methods:**

A cross-sectional, double-blind study was conducted among fourth-year Bachelor of Dental Surgery students at King Khalid University. Forty-five students prepared the maxillary right central incisor for a metal-ceramic crown under standardized conditions. Preparations were independently evaluated by four calibrated prosthodontists using an analytic rubric comprising eight criteria, each scored on a 0–1 scale. Descriptive statistics, one-way ANOVA, and *post hoc* Tukey's HSD were employed to analyze examiner variation. Inter-rater reliability was assessed using Cohen's kappa.

**Results:**

The overall mean score across examiners was 3.436 ± 0.522. Among the parameters, incisal reduction (57.5%) and degree of taper (61.8%) recorded the lowest scores, while finish line form (76.9%) and gingival protection (76.4%) showed the highest. ANOVA indicated no significant differences among the four examiners across all parameters (*p* > 0.05). Tukey's HSD confirmed no pairwise examiner differences, demonstrating consistent grading. Inter-rater reliability yielded kappa values ranging from 0.67 to 0.85, indicating substantial to almost perfect agreement.

**Conclusion:**

The analytic rubric proved to be a reliable and transparent tool for evaluating preclinical crown preparations, minimizing examiner subjectivity and enhancing feedback.

## 1 Introduction

The foundation of clinical competence in dentistry is established during preclinical training, where students acquire and refine critical psychomotor skills (1). In restorative dentistry and prosthodontics, tooth preparation represents a fundamental skill that must be performed with precision, understanding, and adherence to biomechanical and esthetic principles. The All-ceramic crowns are most recommended esthetic option for anterior tooth but comes with a high cost. So, the next best available option is metal-ceramic crown, which provide esthetics and strength along with cost effectiveness. These metal-ceramic restorations require meticulous execution, combining biomechanical integrity, biological considerations, and esthetic outcomes. Given the irreversible nature of tooth preparation and its direct impact on long-term treatment success, the pedagogical strategies surrounding its instruction and assessment demand scrutiny and refinement (2, 3).

Traditional approaches to evaluating practical skills in dentistry have often relied heavily on subjective methods such as global rating scales, numeric scoring, or general impressions of performance. These traditional approaches often lack specificity and standardization, which can lead to variability in scoring, reduced reliability among evaluators, and limited formative feedback for learners (4, 5). The need for structured, objective, and pedagogically sound assessment tools has therefore prompted the exploration of analytic rubrics—tools designed to deconstruct complex clinical procedures into discrete, measurable components. Analytic rubrics have emerged as a promising alternative to conventional assessment methods (1, 2, 6). A rubric provides a matrix of defined criteria and performance levels, enabling assessors to evaluate specific elements of a task in a transparent and structured manner. In the context of crown preparation, an analytic rubric can dissect the task into detailed parameters such as incisal and axial reduction, taper, margin placement, and soft tissue management (5, 7, 8). This detailed breakdown facilitates not only objective grading but also offers rich feedback to guide student learning and self-assessment. Thus, rubrics facilitate clearer expectations, more consistent evaluations, and deeper student understanding of performance standards, especially in skill-centric disciplines like prosthodontics (1, 9, 10).

This present study was conducted within the framework of a meticulously structured course titled “Introduction to Fixed Prosthodontics”, delivered to undergraduate (UG) level 7 students (corresponding to fourth year) Bachelor of Dental Surgery (BDS) students at King Khalid University. As a cornerstone of preclinical training, this course introduces students to the foundational principles of crown and bridge prosthodontics, emphasizing hands-on mastery of individual crown preparations, impression techniques, and provisional restorations (10–12). The course design integrates theoretical instruction with extensive laboratory-based practical sessions, ensuring that students are not only conceptually informed but also experientially competent.

In this context, evaluating the anterior metal-ceramic crown preparation provides a lens through which to assess both student performance and the effectiveness of the analytic rubric as an assessment instrument. The anterior crown preparation, especially on the maxillary right central incisor, presents unique challenges. It demands precision in incisal reduction, facial and lingual axial contouring, and harmonious convergence angles, all while preserving periodontal health and respecting adjacent tooth anatomy. These technical demands are compounded by the aesthetic considerations inherent to the anterior zone, thereby making the exercise an ideal benchmark for assessing preclinical competence (6, 13).

Recognizing the limitations of traditional assessment methods, the study implemented an analytic rubric composed of ten parameters, each focusing on a distinct aspect of the preparation process. The rubric's design was informed by both educational theory and best practices in prosthodontic training, drawing upon authoritative texts such as Fundamentals of Fixed Prosthodontics by Shillingburg and Contemporary Fixed Prosthodontics by Rosenstiel, Land, and Fujimoto (5, 14, 15).

Moreover, the rubric's application was not isolated from pedagogical intent. Its use served dual purposes: first, to enable fair and objective summative evaluation; and second, to guide formative feedback that could support student reflection, self-assessment, and iterative skill improvement (9, 16). These aspects are essential in the context of modern dental education, where emphasis is increasingly placed on developing lifelong learners equipped with self-regulatory abilities and critical thinking skills (7).

The alignment of the rubric with the course learning outcomes—particularly those coded under the skills domain—ensured that the assessment captured essential competencies such as demonstration of proper tooth reduction, understanding margin configurations, and manipulation of dental materials (13, 14, 16). Additionally, the rubric supported the course's value-based outcomes, reinforcing professional behaviors such as neatness, discipline, and ergonomic practice.

Globally, various studies have been conducted in medical and dental curricula. Studies by Satheesh et al. (14), Escribano et al. (7), and Abiad (15) have highlighted the reliability and educational benefits of rubric-based assessments in periodontics, endodontics, and operative dentistry, respectively. In prosthodontics, however, much of the literature has focused on posterior teeth or all-ceramic restorations (5), with limited exploration into anterior PFM preparations—an important gap this study seeks to address.

Furthermore, the rubric's implementation responds to calls for evidence-based assessment practices in education frameworks. It supports the guidelines set forth by accreditation bodies and curriculum committees, which emphasize measurable learning outcomes, transparency in evaluation, and continuous quality improvement (17). The course under consideration in the present study embodies these principles through its inclusion of continuous assessments, and structured daily evaluations, creating a conducive environment for integrating the rubric as both an instructional and assessment tool.

The anterior metal-ceramic crown preparation presents unique challenges due to its dual aesthetic-functional demands and technical intricacies, necessitating a specialized approach to evaluation (8, 18). This study was conducted to fill the research gap in current literature by investigating the application of a comprehensive analytic rubric system for assessing anterior metal-ceramic crown preparations done by UG dentistry students.

This study sought to examine the application of an analytic rubric system for assessing metal-ceramic crown preparations on the maxillary central incisor performed by dental students. The further objective was to examine the rubric's effectiveness in distinguishing performance quality. By doing so, this study aims to contribute to the refinement of competency-based assessment in dental education and to support educators in implementing reliable, valid, and pedagogically beneficial evaluation methods.

## 2 Materials and methods

### 2.1 Study design and setting

This cross-sectional double blinded study was conducted at the Department of Prosthetic dentistry, College of Dentistry, King Khalid University. The Ethical clearance was obtained (IRB/KKUCOD/ETH/2024-25/031) from the ethical committee of the College of Dentistry.

### 2.2 Participants and inclusion, exclusion criteria

Fourth-year undergraduate students enrolled in a preclinical fixed prosthodontics course (academic year 1445-46-Semester -1; corresponds to 2024-25) were evaluated. Students who had successfully fulfilled all prerequisite courses, met all course requirements, and provided consent for the utilization of their data were incorporated into the study. Conversely, students who did not complete the required preparation within a stipulated duration of 45 min or who altered their work outside the designated phantom head setup were excluded from participation and were asked to redo the procedure. A written informed consent was secured from all students prior to their involvement in this research endeavor. Participants were duly informed that their engagement was entirely voluntary and that they possessed the right to withdraw at any point without any repercussions on their academic standing.

### 2.3 Course overview and structure

The anterior metal-ceramic crown preparation evaluated in this study was conducted as a core component of the course titled “Introduction to Fixed Prosthodontics - I”, offered to fourth-year students (7th level) enrolled in the Bachelor of Dental Surgery (BDS) program at King Khalid University, College of Dentistry. This course is delivered under the Department of Prosthetic Dentistry and forms an essential element of the college's preclinical curriculum.

“Introduction to Fixed Prosthodontics - I” is a 3-credit hour course (1 theory+2 practical) designed to provide foundational training in the concepts, design principles, and hands-on execution of fixed prosthodontic restorations. The students are taught using a hybrid mode consisting of classroom instruction (16 h) and extensive laboratory training (64 h), totalling 80 contact hours. The **c**urriculum content includes various basic topics including principles of tooth preparation and metal ceramic crown preparation in anterior and posterior tooth.

### 2.4 Teaching and assessment methods

The instructional strategy integrates didactic lectures, group discussions, hands-on lab sessions, and e-learning modules. The course utilizes high-quality instructional resources including: Lecture rooms with modern multimedia equipment; Dental simulation labs equipped with phantom heads with associated instruments. The primary textbooks recommended in the course included *Fundamentals of Fixed Prosthodontics* by Shillingburg et al. and *Contemporary Fixed Prosthodontics* by Rosenstiel et al. Additional resources include digital content through the university's e-learning portal and access to recent review articles on restorative protocols.

The course emphasizes final practical examination and adopts an outcome-driven approach. Assessment methods included-Mid-term MCQ-based examination, Continuous assessment based on lab performance and professionalism, Final practical examination and E-learning quizzes and final theory exams. Students perform crown preparations on typodont teeth under guided supervision, using standardized equipment and materials. Daily assessments were conducted during practical sessions to provide formative feedback and monitor each student's skill progression. Performance is evaluated using detailed rubrics for specific tasks, in the present study the metal ceramic crown was assessed using pre-decided Rubrics.

### 2.5 Procedure

Students prepared a maxillary right central incisor (tooth #11) for a metal-ceramic crown on a typodont model (Frasaco, Tettnang, Germany), mounted in a phantom head. All students followed a standardized instruction session and were given 45 min to complete the preparation. Teeth were de-identified and randomized for blind evaluation (5). The contributing male and female students were given clear guidelines regarding the exercise type and were informed about the analytic rubric criteria for the assessment; they also exercised in the same manner earlier during practice in the same academic year.

### 2.6 Analytic rubric design

Evaluation was performed using a structured analytic rubric that aligned directly with the course objectives and competency standards, ensuring consistency in grading and alignment with expected educational outcomes. The assessment sheets with rubric criteria were given to students at the start of semester. Total 10 marks were allotted for the Metal ceramic crown exercise. The 50% marks i.e., 5 marks were for the hand skill assessment; 30% marks i.e., 3 marks were for knowledge assessment and 20% i.e., 2 marks were for professionalism. This study was concentrated to assess the hand skills.

The evaluative criteria for the assessment of manual dexterity were established through an extensive examination of pre-existing assessment rubrics documented in the scholarly literature, complemented by discussions with faculty members specializing in prosthodontics within the department. The criteria were designed to thoroughly evaluate essential components of metal ceramic crown fabrication. The rubric framework underwent a validation process via pilot testing with faculty members who were not directly engaged in the research, and their insights, alongside those from students, were integrated into the final iteration; the reliability of the rubric system was substantiated through inter-rater agreement, as both evaluators independently assessed the same collection of prepared specimens, and their scores were subsequently compared, revealing a high degree of reliability. Based on the results, minor adjustments were made to ensure alignment.

The rubric included 8 criteria: 1-Axial wall reduction -facial (two plane)/palatal axial reduction 2- Axial wall reduction -mesial/distal axial taper, 3-Incisal reduction, 4-Finish line configuration -Finish line form 5- Finish line location and continuity, 6- Degree of taper (total occlusal convergence), 7- Protecting adjacent tooth, 8- Gingival protection.

**Figure 1:**
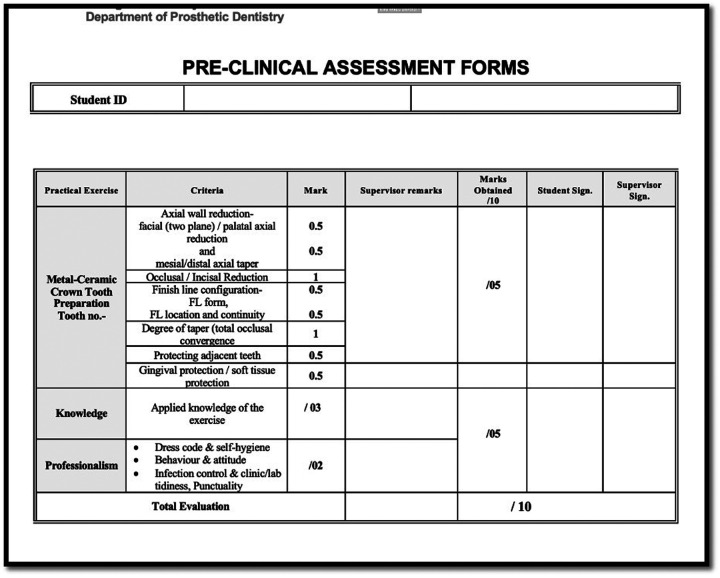
Representative analytical rubric assessment form used in study.

The evaluation of each parameter was further categorized into a maximum score of 1, 0.5, and a minimum score of 0. This analytical rubric employed a grid format wherein the parameters were enumerated in the leftmost column, while the levels of scoring (performance) were articulated across the row utilizing numerical values along with descriptive labels. Each criterion (parameter) was assessed independently, and the rightmost column was filled with the specific score corresponding to each parameter; subsequently, the aggregate of all scores for each parameter was calculated to derive the total score for the individual student (Figure 1). Individual printed sheets were used for the assessment of each student, which were coded (university student ID) according to the identifiers assigned to the typodont jaws for all participating students. For each student, both male and female, the aggregate score was determined by totalling the scores across all criteria.

### 2.7 Evaluation process

Four experienced prosthodontists, calibrated prior to the study, independently evaluated the preparations. Calibration involved reviewing 5 sample preparations and resolving discrepancies through consensus (19). The evaluators were asked to do the evaluation independently with no time restriction.

### 2.8 Statistical analysis

Statistical analysis of the acquired data was conducted utilizing SPSS version 21 (SPSS, Inc., Chicago, IL, USA), with a predetermined significance threshold set at *P* < 0.05. Descriptive statistics were computed for all four examiners as well as for the eight parameters under investigation. A one-way analysis of variance was employed to facilitate the comparison of scores across the eight parameters. To evaluate interexaminer variation, a *post hoc* Tukey test was administered to compare the individual scores for each parameter among all four evaluators.

## 3 Results

**Figure 2:**
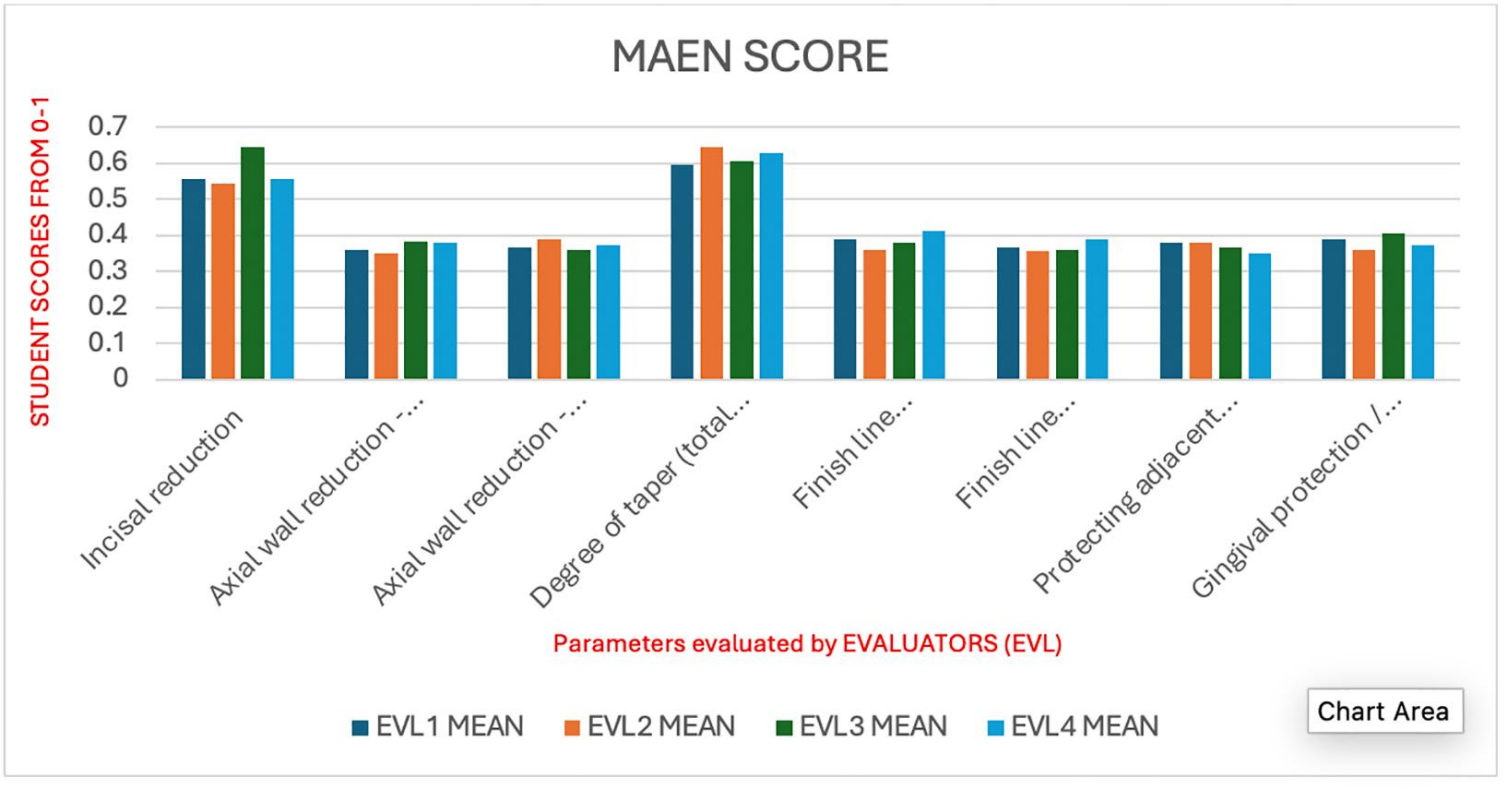
Mean score of parameters for metal ceramic crown preparation by the evaluators.

The parameters critical for the preparation of tooth intended for metal-ceramic crowns were meticulously assessed in the present study. Four evaluators conducted assessments of these parameters utilizing analytical rubrics for a cohort of 45 dental students in their fourth year, enrolled in a pre-clinical fixed prosthodontics course. The descriptive statistics pertaining to the scores assigned to students by the four evaluators are delineated in Table 1. A one-way analysis of variance revealed no statistically significant differences among the examiners across all evaluated parameters. The cumulative mean score across all evaluators was determined to be 3.436 ± 0.522, with the highest and lowest mean scores recorded for Examiner 3 (3.506 ± 0.421) and Examiner 2 (3.383 ± 0.550), respectively, as illustrated in Table 1 and Figure 2.

**Table 1:** Mean (standard deviation-SD) of parameters for metal ceramic crown preparation by the evaluators.

Parameter	EVL1		EVL2		EVL3		EVL4		Overall		ANOVA	
	Mean	SD	Mean	SD	Mean	SD	Mean	SD	Mean	SD	F-value	*p-value
Incisal reduction	0.556	0.286	0.544	0.293	0.644	0.284	0.556	0.301	0.575	0.291	1.15	0.3303
Axial wall reduction - facial (two plane)/palatal axial reduction	0.361	0.126	0.350	0.124	0.383	0.126	0.378	0.126	0.368	0.126	0.669	0.5723
Axial wall reduction - mesial/distal axial taper	0.367	0.126	0.389	0.126	0.361	0.126	0.372	0.126	0.372	0.126	0.409	0.747
Degree of taper (total occlusal convergence)	0.594	0.288	0.644	0.279	0.606	0.258	0.628	0.304	0.618	0.283	0.281	0.8387
Finish line configuration - finish line form	0.389	0.126	0.361	0.126	0.378	0.126	0.411	0.121	0.385	0.125	1.273	0.2852
Finish line configuration - finish line location and continuity	0.367	0.126	0.356	0.125	0.361	0.126	0.389	0.126	0.368	0.126	0.609	0.6098
Protecting adjacent tooth	0.378	0.126	0.378	0.126	0.367	0.126	0.350	0.124	0.368	0.126	0.491	0.6891
Gingival protection/soft tissue protection	0.389	0.126	0.361	0.126	0.406	0.123	0.372	0.126	0.382	0.125	1.088	0.3557
Total of 8	3.400	0.560	3.383	0.550	3.506	0.421	3.456	0.557	3.436	0.522		

Evaluator [EVL] (*n* = 45).

* *p*-values > 0.05, there are no statistically significant differences among the four evaluators (EVL1–EVL4) for any of the scoring parameters.

Tables 2 present the results of *post hoc* Tukey's test, which was applied for the multiple comparisons of the examiners within each parameter. For all eight parameters, ANOVA results were non-significant (*p* > 0.05), indicating no strong statistical evidence of differences among the four examiners. Tukey's HSD confirmed that no examiner pair had significantly different scoring for any parameter. Examiner means were closely aligned, suggesting a high level of inter-examiner consistency. This shows that examiners were well-calibrated, and student evaluations were consistent across different assessors.

**Table 2:** Results of *post hoc* Tukey's test comparing the score of 4 examiners within each parameter.

Parameter	Evaluator- A	Evaluator-B	Mean Diff	*P*-Adj	CI lower	CI upper
Axial wall reduction - facial (two plane)/palatal axial reduction	EVL-1	EVL-2	−0.011	0.975	−0.080	0.058
	EVL-1	EVL-3	0.022	0.835	−0.046	0.091
	EVL-1	EVL-4	0.017	0.922	−0.052	0.085
	EVL-2	EVL-3	0.033	0.590	−0.035	0.102
	EVL-2	EVL-4	0.028	0.720	−0.041	0.096
	EVL-3	EVL-4	−0.006	0.997	−0.074	0.063
Axial wall reduction - mesial/distal axial taper	EVL-1	EVL-2	0.022	0.837	−0.047	0.091
	EVL-1	EVL-3	−0.006	0.997	−0.074	0.063
	EVL-1	EVL-4	0.006	0.997	−0.063	0.074
	EVL-2	EVL-3	−0.028	0.723	−0.097	0.041
	EVL-2	EVL-4	−0.017	0.923	−0.086	0.052
	EVL-3	EVL-4	0.011	0.975	−0.058	0.080
Degree of taper (total occlusal convergence)	EVL-1	EVL-2	0.050	0.837	−0.105	0.205
	EVL-1	EVL-3	0.011	0.998	−0.144	0.166
	EVL-1	EVL-4	0.033	0.944	−0.122	0.188
	EVL-2	EVL-3	−0.039	0.915	−0.194	0.116
	EVL-2	EVL-4	−0.017	0.992	−0.172	0.138
	EVL-3	EVL-4	0.022	0.982	−0.133	0.177
Finish line configuration - finish line form	EVL-1	EVL-2	−0.028	0.716	−0.096	0.040
	EVL-1	EVL-3	−0.011	0.975	−0.079	0.057
	EVL-1	EVL-4	0.022	0.833	−0.046	0.090
	EVL-2	EVL-3	0.017	0.921	−0.052	0.085
	EVL-2	EVL-4	0.050	0.231	−0.018	0.118
	EVL-3	EVL-4	0.033	0.584	−0.035	0.102
Finish line configuration - finish line location and continuity	EVL-1	EVL-2	−0.011	0.975	−0.080	0.058
	EVL-1	EVL-3	−0.006	0.997	−0.074	0.063
	EVL-1	EVL-4	0.022	0.836	−0.046	0.091
	EVL-2	EVL-3	0.006	0.997	−0.063	0.074
	EVL-2	EVL-4	0.033	0.590	−0.035	0.102
	EVL-3	EVL-4	0.028	0.721	−0.041	0.096
Gingival protection/soft tissue protection	EVL-1	EVL-2	−0.028	0.718	−0.096	0.041
	EVL-1	EVL-3	0.017	0.922	−0.052	0.085
	EVL-1	EVL-4	−0.017	0.922	−0.085	0.052
	EVL-2	EVL-3	0.044	0.334	−0.024	0.113
	EVL-2	EVL-4	0.011	0.975	−0.057	0.080
	EVL-3	EVL-4	−0.033	0.587	−0.102	0.035
Incisal reduction	EVL-1	EVL-2	−0.011	0.998	−0.170	0.148
	EVL-1	EVL-3	0.089	0.472	−0.070	0.248
	EVL-1	EVL-4	0.000	1.000	−0.159	0.159
	EVL-2	EVL-3	0.100	0.366	−0.059	0.259
	EVL-2	EVL-4	0.011	0.998	−0.148	0.170
	EVL-3	EVL-4	−0.089	0.472	−0.248	0.070
Protecting adjacent tooth	EVL-1	EVL-2	0.000	1.000	−0.069	0.069
	EVL-1	EVL-3	−0.011	0.975	−0.080	0.058
	EVL-1	EVL-4	−0.028	0.721	−0.097	0.041
	EVL-2	EVL-3	−0.011	0.975	−0.080	0.058
	EVL-2	EVL-4	−0.028	0.721	−0.097	0.041
	EVL-3	EVL-4	−0.017	0.923	−0.085	0.052

Based on evaluator means, the largest spread in average scores across evaluators was observed for Incisal reduction. Evaluator-3 had a notably higher mean (0.644) compared to Evaluator-2 (0.544) and Evaluator-1/4 (0.556 each). Although ANOVA was not statistically significant (*p* = 0.3303), this parameter displayed the largest observed variation in mean scoring patterns. The lowest variation in scoring was found for Finish line configuration – finish line form (finishing of margins and walls of the preparation). Evaluator means ranged narrowly between 0.361 and 0.411. ANOVA confirmed no significant differences (*p* = 0.2852), supporting very high consistency among evaluators for this parameter.

Overall, the findings highlight that although the examiners showed high consistency, students' average performance varied across parameters. Lower mean scores were particularly evident in incisal reduction – 0.575 and degree of taper – 0.618, suggesting that students experienced the greatest difficulty with these aspects of tooth preparation. Gingival protection/soft tissue protection – 0.764 and finish line configuration (margins and walls) – 0.769 had the highest average scores and the least inter-examiner variation, reflecting strong student performance and examiner agreement in this area. The pairwise kappa values between the examiners ranged from 0.67 to 0.85, indicating a substantial to almost perfect agreement according to Landis and Koch's benchmarks. The kappa matrix demonstrated strong reliability across all examiner pairs, with no value falling below 0.67. It was evident that while examiner variability was minimal and non-significant, students require targeted improvement in incisal reduction and degree of taper. These areas represent critical aspects of crown preparation where skill reinforcement is most needed. The other parameters of preparation were generally scored above average and can be regarded as satisfactory.

## 4 Discussion

This study provides comprehensive insights into the use of an analytic rubric system for evaluating anterior metal-ceramic crown preparations, emphasizing its significance in enhancing assessment reliability, promoting pedagogical transparency, and identifying performance variances among dental students. The findings support the hypothesis that rubric-based evaluations offer a superior alternative to traditional assessment modalities in dental education.

One of the most critical aspects of prosthodontic education is teaching students the meticulous techniques of tooth preparation, particularly for metal-ceramic restorations, which demand both functional integrity and aesthetic accuracy (20). The development and validation of an analytic rubric allowed for detailed analysis of individual components of student performance—incisal reduction, axial wall taper, margin design, finish line location and continuity, among others. This breakdown provided students with specific feedback on their strengths and areas for improvement (17, 21–23).

In the field of dentistry, the implementation of a criterion-oriented grading system has been established for over four decades. Dhuru et al. (24) in 1978 highlighted the significance of, and advocated for the adoption of, criterion-oriented grading within pre-clinical dentistry curricula. The efficacy of this criteria-based assessment approach has been corroborated by numerous researchers in subsequent years (25). Given the constrained availability of resources and access to more sophisticated digital assessment tools in a majority of dental schools globally, the significance of the analytic rubric in pre-clinical dentistry courses cannot be overstated. Nevertheless, the application of rubrics demands a greater investment of time when compared to the global grading methodology, and in the absence of precise definitions for each criterion pertaining to individual parameters within the rubrics, the likelihood of inter-examiner variability in scoring remains elevated.

The observed kappa values demonstrate that the four examiners were generally well calibrated in their application of the rubric. The range of 0.67–0.85 confirms that inter-rater reliability was robust**,** minimizing the potential impact of subjectivity on student evaluations. Although Examiner-4 displayed slightly lower agreement with Examiner-1, the overall pattern indicates that examiners applied the scoring criteria with a high degree of consistency. This finding supports the effectiveness of the analytic rubric used in this study for standardizing assessment across multiple evaluators (14, 25, 26). This consistency is vital in preclinical settings where uniformity in assessment contributes to fair grading and better student understanding of performance expectations (2, 9, 13).

Despite this, certain parameters such as incisal reduction showed only fair agreement, echoing challenges noted in prior studies (27–29). The interpretation of subtle anatomical nuances, particularly on typodont models without digital augmentation, may account for lower agreement in visual evaluations (17, 20, 28). This highlights a potential area for improvement in assessment practices—specifically, incorporating digital or CAD/CAM-based evaluation tools to supplement visual grading (9, 17). These technologies offer 3D measurement capabilities that can precisely assess convergence angles, margin depths, and incisal reductions, thereby minimizing human error (30, 31).

In the present study the gender-based analysis was not performed as hand skills are not affected gender and well supported by the previous work of Kornmehl et al. (21), who noted minimal gender discrepancies in objective skill-based assessments. However, other studies report variations potentially influenced by learning styles, instructor bias, or student self-perception (21, 27, 30, 32).

Rubrics have been shown to increase student engagement and confidence by setting transparent performance benchmarks (7, 10, 15, 17). When rubrics are introduced early in the curriculum, students become more adept at self-assessment—a competency increasingly emphasized in modern dental education frameworks (7, 9, 10).

It was hypothesized that the use of analytic rubrics could improve the consistency of scoring across the four examiners. The results of this study showed an overall mean performance of 72.3% across all parameters for the participating students. For most of the examiners, their average scores were closely aligned with this overall mean, reflecting a high level of calibration. A slight tendency to diverge was observed for Examiner 3 (64.4% in incisal reduction), who graded somewhat lower in that parameter, while Examiner 2 (74.4% in mesial/distal taper) scored marginally higher than the others. Nonetheless, the differences among examiners were not statistically significant, as confirmed by one-way ANOVA (*p* > 0.05) and Tukey's HSD pairwise comparisons. The maximum observed difference was between Examiner 3 (64.4%) and Examiner 2 (74.4%), with a mean gap of approximately 10 percentage points, which is still within acceptable limits of variability. These results demonstrate that while minor variations in examiner scoring exist, overall, there was good inter-examiner consistency in the use of the rubric. This finding is consistent with prior reports in the literature that rubric-based assessment frameworks increase scoring reliability and reduce subjective bias in clinical evaluation (9, 13, 18).

The analytical rubric system facilitates the provision of comprehensive feedback to students, thereby enhancing their capacity to derive extensive insights from their assessments regarding each specified criterion (33, 34). This mechanism allows students to accurately identify their specific areas of strength and weakness through the provision of detailed results, eliminating the necessity for explicit commentary from their instructors (33). In the context of the individual preparation parameters examined in the present study, the criteria in which students exhibited the lowest performance were incisal reduction and degree of taper, both of which are pivotal to the long-term efficacy of metal-ceramic crowns. These errors have implications beyond academic grading—they affect retention, resistance, and esthetics of the final restoration. As such, repeated reinforcement and focused skill sessions on these parameters may be warranted. Utilizing vertical depth grooves or reduction guides may help students better control their reduction dimensions (8, 29).

Appropriate axial reduction is essential to ensure sufficient space for facilitating optimal functional morphology and structural durability. Vertical depth grooves are executed on the vestibular surface utilising a tapered diamond bur, subsequently followed by the preparation of the tooth structure situated between the grooves. The establishment of depth grooves serves to regulate the extent of reduction. Rosella et al. (35) articulated that these parameters represent the most significant challenge for prosthodontists, specifically in terms of managing the depth and trajectory of dental tissue excision.

Several previous investigations have underscored the difficulty in mastering appropriate taper and uniform reduction. Habib (3) and Mobarki et al. (36) both reported high variability in students' taper angles and finish line execution. These challenges, also seen in our findings, highlight the importance of regular formative feedback supported by clear, criterion-based rubrics.

Emerging evidence also advocates for integrating digital simulation with traditional exercises. Studies by Han et al. (28) and Miyazono et al. (36) demonstrate the benefits of augmented reality and 3D evaluation platforms in enhancing skill acquisition and assessment objectivity. While our study relied solely on visual inspection, the use of digital adjuncts in future studies could further improve precision and reproducibility.

Lastly, while this study focuses on evaluating a single practical exercise, the potential applications of analytic rubrics extend far beyond crown preparation. Rubrics have been successfully implemented in orthodontic presentations (16), periodontic competency exams (14), and endodontic skill evaluations (15). Their broader integration into dental curricula may represent a paradigm shift toward competency-based education that values clarity, feedback, and objective measurement.

The primary aim of pre-clinical dental education is to equip students adequately for the provision of optimal dental care to their patients within clinical settings. Subsequently, the students augment the foundational knowledge acquired in the pre-clinical courses, with the learning process continuing throughout their more advanced clinical training. A research study conducted by Velayo et al. (37) demonstrated a positive correlation between pre-clinical performance and subsequent clinical success. Students identified as lacking in practical competencies may engage in supplementary practice to enhance their psychomotor skills (38).

The application of an analytic rubric in evaluating anterior metal-ceramic crown preparations offers compelling evidence for its adoption in dental education. It improves inter-rater reliability, enhances feedback quality, encourages student engagement, and aligns with modern educational outcomes. However, complementary use of digital technologies and continuous rubric refinement will be essential to maximize their academic value. Similar types of rubrics can generated for every course with modifications as per the exercise.

### 4.1 Limitations

It is worth noting the study's limitations. Despite evaluator calibration, slight bias cannot be ruled out. It was conducted at a single institution with a limited sample size, which may restrict the generalizability of the findings. The evaluation focused solely on the maxillary right central incisor prepared for a metal-ceramic crown; hence, results may not apply to other tooth types or restoration designs. Although examiner calibration was performed, minor subjective bias in visual assessment cannot be entirely excluded. Additionally, the absence of digital or CAD/CAM-assisted evaluation limited precision in measuring preparation parameters. The cross-sectional nature of the study also precluded assessment of longitudinal learning outcomes and student perceptions of rubric-based feedback.

Future studies should involve larger, multi-institutional samples and incorporate digital assessment tools to enhance accuracy and reproducibility. Longitudinal research exploring the impact of rubric-guided evaluation on student learning, self-assessment skills, and clinical performance is also recommended to further validate and expand the utility of analytic rubrics in dental education. Also, qualitative data (student feedback, examiner perceptions) must also be assessed.

## 5 Conclusion

The application of an analytic rubric for evaluating anterior metal-ceramic crown preparation offers a consistent, reliable, and pedagogically sound approach to student assessment. The analytic rubric employed for crown preparations within the context of pre-clinical fixed prosthodontics serves as a valuable instrument for identifying errors and deficiencies among dental students. Within the parameters of tooth preparation examined in this research, the most prominent areas of weakness identified among the participating students were axial reduction and the resultant damage to the adjacent teeth. Further studies should compare rubric performance with digital evaluation tools and explore longitudinal outcomes.

## Data Availability

The original contributions presented in the study are included in the article/Supplementary Material, further inquiries can be directed to the corresponding author.
